# Neutrophil-to-Lymphocyte Ratio Predicts Early Neurological Deterioration after Endovascular Treatment in Patients with Ischemic Stroke

**DOI:** 10.3390/life12091415

**Published:** 2022-09-10

**Authors:** Simona Lattanzi, Davide Norata, Serena Broggi, Stefano Meletti, Milena Świtońska, Artur Słomka, Mauro Silvestrini

**Affiliations:** 1 Neurological Clinic, Department of Experimental and Clinical Medicine, Marche Polytechnic University, 60121 Ancona, Italy; 2 Neurology Unit, OCB Hospital, AOU Modena, 41125 Modena, Italy; 3 Department of Biomedical, Metabolic and Neural Science, Center for Neuroscience and Neurotechnology, University of Modena and Reggio Emilia, 41121 Modena, Italy; 4 Department of Neurosurgery and Neurology, Nicolaus Copernicus University in Toruń, Ludwik Rydygier Collegium Medicum, Faculty of Health Sciences, 85-067 Bydgoszcz, Poland; 5 Department of Pathophysiology, Nicolaus Copernicus University in Toruń, Ludwik Rydygier Collegium Medicum in Bydgoszcz, 85-067 Bydgoszcz, Poland

**Keywords:** cerebrovascular disease, ischemic stroke, neutrophils, lymphocytes, neutrophil-to-lymphocyte ratio

## Abstract

The worsening of neurological status that occurs early after acute ischemic stroke (AIS) remains a serious issue, and the inflammatory response plays a key role in stroke pathobiology. Recently, endovascular treatment (EVT) has revolutionized the management and outcome of patients with AIS due to either extracranial carotid disease or intracranial disease. The neutrophil-to-lymphocyte ratio (NLR) represents an easily available inflammatory biomarker. The aim of the study was to assess the relationship between the NLR at admission and the occurrence of early neurological deterioration (END) in patients with AIS who underwent EVT. Patients with AIS and proximal arterial occlusion in the anterior circulation undergoing EVT were retrospectively identified. Absolute neutrophil count (ANC) and absolute lymphocyte count (ALC) were collected from admission blood work to calculate the NLR. The study outcome was END defined as an increase in at least 4 points in NIHSS score or death between baseline and 24 h after the ischemic event. Patients included were 211, and END occurred in 30 (14.2%). Patients with older age (OR = 1.07, 95% CI: 1.02–1.13), higher serum glucose (OR = 1.01, 95% CI: 1.01–1.02), and higher NLR (OR = 1.011, 95% CI: 1.04–1.18) had an increased risk of END. The best predictive cut-off value of NLR was 6.4, and END occurred in 24.1% and 3.9% of the patients with NLR ≥ 6.4 and <6.4, respectively (*p* < 0.001). In patients with AIS undergoing EVT, higher NLR values predicted a higher risk of END. Biomarkers able to identify inflammatory mechanisms might identify novel treatment targets and enhance proof-of-concept trials of immunomodulation in stroke.

## 1. Introduction

Stroke is a leading cause of mortality and morbidity worldwide [1]. According to the epidemiological statistics of the global burden of disease, in 2019, there were 12.2 million incident cases and 101 million prevalent cases of stroke, with 6.55 million and 143 million disability-adjusted life years due to stroke [2]. Globally, stroke remains the second-leading cause of death and the third-leading cause of death and disability combined. Of note, the age-standardized rates of stroke-related mortality and stroke-related disability-adjusted life years were 3.6–3.7 times higher in the low- than high-income countries [2].

Ischemic stroke constitutes around 60–65% of all incident strokes. Although the endovascular treatment (EVT) has been demonstrated to represent a key therapy to improve clinical outcome, the rate of death and functional dependence is still high [3,4,5,6]. Importantly, the worsening of acute stroke early in its course remains a serious issue associated with poor outcome. Early neurological deterioration (END) has been reported to occur in 10% to 40% of stroke patients, and the differences in diagnostic criteria of clinical worsening, timing of assessment, and case mix of patients can account for the wide range in incidence [7]. Initial stroke severity has been shown to be a strong independent predictor of END [7,8]. Among biochemical biomarkers, high serum glucose levels and high high-sensitivity C-reactive protein were associated with clinical deterioration [7,9,10,11]. Low Alberta Stroke Program Early Computed Tomography Score (ASPECTS), poor collaterals, large vessel occlusion, extent of hypodensity > 33% in the middle cerebral territory, hyperdense middle cerebral artery sign, and cerebral edema on early brain CT may reflect severe initial stroke and have been found to be radiological predictors [12,13,14]. Age, history of diabetes mellitus, hypertension, and prestroke modified Rankin Scale score ≥ 2 were also recognized as risk factors for END in some studies, whereas the independent role of baseline blood pressure levels has not been established [8,12,13,15,16]. General anesthesia, unsatisfactory recanalization of occluded arteries, and number of EVT passes can further affect the risk of neurological worsening [8,12]. Thus far, only a few studies have investigated the occurrence and predictors of END in stroke patients undergoing EVT [8,13,17,18].

The inflammatory response plays a key role in the pathophysiology of ischemic stroke, and it has been shown to be involved in the secondary progression of ischemic lesion [11,19,20]. In this regard, the neutrophil-to-lymphocyte ratio (NLR), which is easily calculated from the neutrophil and lymphocyte counts, represents a reliable and inexpensive measure of the inflammatory levels, and it is recently gaining attention as a prognostic biomarker in the field of acute cerebrovascular diseases. Previous studies have reported that the NLR may be a good predictor of mortality and short-term functional status, angiographic outcomes, intracerebral hemorrhage, and stroke-associated infection or pneumonia in patients with acute ischemic stroke, including stroke patients who receive reperfusion therapies [21,22,23,24,25,26,27,28,29,30]. Interestingly, the significant associations found between the NLR and the outcome of patients with either intracerebral hemorrhage or subarachnoid hemorrhage suggest how commonalities may exist in the inflammatory pathways underlying stroke-induced secondary brain damage [31,32,33].

The aim of this study was to evaluate the relationship between the NLR at admission and the occurrence of END in patients with acute ischemic stroke (AIS) undergoing EVT.

## 2. Methods

### 2.1. Study Participants

In this retrospective cohort study, consecutive patients with AIS admitted (January 2016 to December 2019) at the Neurology Unit of the Marche Polytechnic University (Ancona, Italy) and treated with intravenous thrombolysis (IVT) plus EVT or EVT alone were identified. Patients were included if they had intracranial proximal arterial occlusion in the anterior circulation (i.e., intracranial carotid artery or M1/M2 segments of middle cerebral artery) and were treated with IVT within 4.5 h and EVT within 6.0 h after the stroke onset according to national and international stroke guidelines [34,35,36,37]. Endovascular treatment included mechanical thrombectomy with aspiration catheters alone, stent retrievers alone, or both, depending on occlusion type/location and the neurointerventionist’s choice; emergency carotid stent placement was decided by the treating neurointerventionist [38,39]. Demographics, clinical history, initial stroke severity by the National Institutes of Health Stroke Scale (NIHSS) score [40], and extension of ischemic lesion by the ASPECTS [41] were retrieved, as previously detailed [42,43,44]. Total white blood cells, absolute neutrophil count (ANC), and absolute lymphocyte count (ALC) were collected from admission blood work within 24 h after stroke onset. The outcome measure was the END, defined as an increase of at least 4 points in NIHSS score or death between baseline and 24 h after the ischemic event [8]. Patients without laboratory values and/or data about 24 h neurological status available were excluded.

### 2.2. Statistical Analysis

Continuous variables were summarized as mean ± standard deviation (SD) or median (quartile deviation(QD)), and categorical variables were presented as the number (%) of patients. The student t-test, Mann–Whitney test, or chi-squared test were used for univariate comparisons; the nonparametric Mann–Whitney test was used when the data did not have a normal distribution. Logistic regression was used to explore the relationship between the NLR and END. The variables with *p*-values < 0.05 from univariate analyses and associations with biologically plausible characteristics (i.e., age, sex, baseline NIHSS score, ASPECT score, and initial serum glucose [7]) were forced in the multivariate model. The ability of the NLR to predict the END was estimated through the receiver operating characteristic (ROC) analysis. The value with the highest Youden’s index was identified as the threshold able to better distinguish the presence of END [45]. Statistical significance was set at *p*-values < 0.05. STATA/IC 13.1 statistical package (StataCorp LP, College Station, TX, USA) was used to perform statistical analysis.

## 3. Results

Patients included in the study were 211, and END occurred in 30 (14.2%) cases. The age of the study cohort was 74 [(10.5)] years, and 101 (47.9%) were men. Eighty-three patients underwent EVT alone, and 128 patients were treated with IVT plus EVT. Details about the procedures of mechanical thrombectomy were available for 205 patients: stent retrievers, aspiration catheters, and a combination of stent retrievers and aspiration catheters were used in 104, 65, and 36 patients, respectively. Carotid artery stent was placed in 21 patients; there was no statistically significant difference in the NLR values according to the stent placement (*p* = 0.311). The baseline characteristics of the participants with and without END are summarized in Table 1. The END group was older; had lower ASPECTS value; had more commonly intracranial occlusion at the level of the internal carotid artery, internal carotid artery terminus, and M1 segment of the middle cerebral artery; and had higher levels of serum glucose, higher white blood cells and neutrophil counts, lower lymphocyte count, and higher NLR values at admission compared with patients without END.

Age, baseline serum glucose, and admission NLR resulted in independently being associated with END: participants who were older (odds ratio (OR) = 1.07, 95% confidence interval (CI): 1.02–1.13, *p* = 0.005) and had higher serum glucose (OR = 1.01, 95% CI: 1.01–1.02, *p* = 0.002) and higher NLR (OR = 1.011, 95% CI: 1.04–1.18, *p* = 0.001) were at higher risk of END (Table 2). There was no collinearity in the multivariate model (variance inflation factors: 1.06 to 1.29).

**Figure 1:**
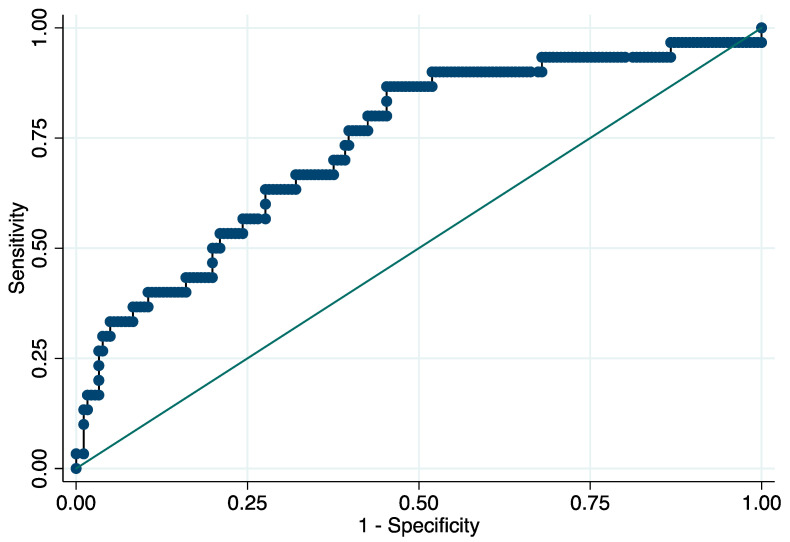
Receiver operating characteristic curve for the prediction of early neurological deterioration. Predictive values of the neutrophil-to-lymphocyte ratio for early neurological deterioration. Area under the curve 0.738 (95% CI: 0.638–0.837). Abbreviation: CI = confidence interval.

At the ROC analysis, the AUC of the NLR for END was 0.738 (95% CI: 0.638–0.837) with 6.4 × 10^9^/L as the best predictive threshold of NLR (sensitivity: 86.7% (95% CI, 69.3–96.2%), specificity: 54.7% (95% CI, 47.1–62.1%), positive predictive value: 24.1% (95% CI, 20.4–28.2%), negative predictive value: 96.1% (95% CI, 90.8–98.4%), positive likelihood ratio (LR): 1.91 (95% CI, 1.55–2.37), negative LR: 0.24 (95% CI, 0.10–0.61)) (Figure 1). Early deterioration of neurological status occurred in 24.1% and 3.9% of the patients with NLR ≥ 6.4 and <6.4, respectively (*p* < 0.001). NLR ≥ 6.4 × 10^9^/L was an independent predictor of END (OR = 7.85, 95% CI: 2.63–23.40, *p* < 0.001; OR_adj_ = 6.68, 95% CI: 2.04–21.92, *p* = 0.002).

## 4. Discussion

The relationship between the NLR and the development of END in patients with AIS treated with EVT represents the main novelty provided by this research. Patients with ischemic stroke and higher NLR at admission were more likely to present early deterioration of the neurological status after stroke, and an NLR value of 6.4 resulted in the best discriminating threshold for the occurrence of END. These results support the increasing body of evidence that inflammatory pathways are crucial determinants of the pathophysiology and prognosis of acute vascular brain injury [46].

Inflammation may contribute to different mechanisms underlying END, including the formation of cerebral edema, progression of infarction, and hemorrhagic transformation [7]. The inflammatory response after stroke is triggered by the release of mediators from damaged brain tissue and develops early after cerebral infarct. Brain-resident macrophages are activated, and blood–brain barrier disruption favors the infiltration of peripheral immune cells into the site of injured tissue [47]. The prominent influx of polymorphonuclear cells and monocytes into the brain increases local inflammation. During the acute phase, leukocytes produce inflammatory cytotoxic mediators that favors cellular injury, promotes capillary permeability, and stimulates prothrombotic pathways that exacerbate edema development, ischemic damage, and secondary progression of brain injury [48,49]. Hemorrhagic transformation, which also represents a sign of endothelial disruption, has been linked to brain leukocyte infiltration [50]. The infiltration of matrix metalloproteinase-9 (MMP-9)-positive neutrophils is associated with the breakdown of the blood–brain barrier and the degradation of basal lamina type IV collagen, which favors hemorrhagic complications [49,51]. The plugging of microvessels that follows endothelial activation, the recruitment of leukocytes, and the aggregation of platelets can impair the reperfusion of tissue and affect the viability of the penumbra area [52,53]. Thromboinflammatory pathways also contribute to the ischemia-reperfusion injury [54]; in this regard, the endothelium–leukocyte–platelet interaction is responsible for microvascular events and secondary thrombosis in the microvasculature that facilitate the growth of infarct size despite the recanalization of large vessels [54].

The insult to the central nervous system is also able to trigger the release of catecholamines and cortisol, which promote the death and functional impairment of peripheral lymphocytes [55]. Of note, the loss of subpopulations of regulatory lymphocytes that counteract the release of proinflammatory mediators and the activation of microglia may compromise the immune homeostasis and cause an imbalance between inflammatory and anti-inflammatory pathways [56,57,58]. In this regard, the SIRI can synthetize the balance between innate and adaptive immunity, with higher values indicating the increased activity of the former and the decreased activity of the latter.

Our data expand the already-accumulated evidence underlying the opportunity to enhance the prediction of the outcome of stroke patients by using serum indices. Of note, an increased peripheral inflammatory response measured by the NLR after stroke reperfusion therapy has been shown to correlate with the occurrence of hemorrhagic transformation and severity of cerebral edema, and a few studies have provided preliminary evidence about its relationship with the worsening of the neurological status [29,59,60]. In this context, it is worth emphasizing that indices incorporating different cellular types can ensure greater accuracy and represent more reliable indices to be used in real-world scenarios [61,62,63,64]. The assessment of a single cell line may be affected by conditions such as dehydration and overhydration and may be less accurate to synthetize the entanglement of the inflammatory pathways.

The real-world setting, which allows us to generalize the results to everyday clinical contexts, and the ready availability and cost effectiveness of the NLR, which is obtained from widely accessible and routinely collected variables, represent major study strengths. Different limits need, however, to be also acknowledged. The retrospective collection of data and the inclusion of patients admitted to one single academic center may have resulted in selection bias. The lack of information about the development of procedural complications, hemorrhagic transformation, and cerebral edema prevented us from exploring the relationship between the NLR and END according to the underlying pathophysiological mechanisms. Further, carotid artery stent was placed in a few patients, and any potential interference between stenting and NLR values could not be definitively explored. The current findings did not allow us to draw definitive conclusions about the causal association between the NLR and END but could stimulate speculations and generate hypotheses. Further studies characterized by larger samples and a prospective design with the assessment of variables such as the infarct volume, status of collaterals, and occurrence of early complications are warranted to confirm and externally validate these findings and provide additional clinical insights.

## 5. Conclusions

The NLR is a low-cost and readily available inflammatory biomarker that could be useful to identify stroke patients at higher risk of END after EVT. Interestingly, several pilot trials explored the role of immunomodulatory agents in ischemic stroke [65,66,67]. In this regard, biomarkers able to identify patients more prone to inflammation might also identify those patients who can benefit the most from such interventions and enhance the proof-of-concept trials of immunomodulation in stroke.

## Figures and Tables

**Table 1:** Baseline characteristics of patients.

	Early Neurological Deterioration		*p*-Value
	No (*n* = 181)	Yes (*n* = 30)	
**Demographics**			
Age (years)	72 (10)	79 (5)	0.002 ^a^
Male sex	87 (48.1)	14 (46.7)	0.887 ^b^
**Clinical history**			
Current smoking	38 (21.0)	6 (20.0)	0.901 ^b^
Hypertension	105 (58.0)	22 (73.3)	0.112 ^b^
Diabetes mellitus	23 (12.7)	3 (10.0)	0.676 ^b^
Dyslipidemia	80 (44.2)	12 (40.0)	0.668 ^b^
Coronary artery disease	30 (16.6)	5 (16.7)	0.990 ^b^
Prior stroke	17 (9.4)	3 (10.0)	0.916 ^b^
**Baseline clinical assessment**			
NIHSS score	15 (3)	14 (2.5)	0.248 ^a^
ASPECT value	9 (1)	8 (1)	0.036 ^a^
Location of intracranial occlusion			0.017 ^b^
Internal carotid artery	23 (12.7)	9 (30.0)	
* Internal carotid artery terminus	9 (5.0)	4 (13.3)	
Middle cerebral artery			
M1 segment	117 (64.6)	13 (43.3)	
M2 segment	32 (17.7)	4 (13.3)	
Serum glucose (mg/dL)	107 (21)	140 (32.5]	<0.001 ^a^
White blood cell count (×109/L)	9660 (2015)	13,580 [3345)	<0.001 ^a^
Absolute neutrophil count (×109/L)	7430 (1980)	11,050 (3795)	<0.001 ^a^
Absolute lymphocyte count (×109/L)	1280 (505)	975 (450)	0.004 ^a^
NLR	5.8 (3.5)	11.8 (6.7)	<0.001 ^a^
**Treatment**			0.375 ^b^
Endovascular treatment alone	69 (38.1)	14 (46.7)	
Intravenous thrombolysis plus endovascular treatment	112 (61.9)	16 (53.3)	

Data are presented as median (QD) for continuous variables, and n (%) for categorical variables. * Associated internal carotid artery and middle cerebral artery occlusion (tandem occlusion). ^a^ Mann–Whitney test. ^b^ Chi-squared test. Abbreviations: ASPECT = Alberta Stroke Program Early CT, NIHSS = National Institutes of Health Stroke Scale, QD = quartile deviation, SD = standard deviation, NLR = neutrophil-to-lymphocyte ratio.

**Table 2:** Association between baseline characteristics and early neurological deterioration.

Dependent Variable	* Adjusted OR (95% CI)	*p*-Value
Age	1.07 (1.02–1.13)	0.005
Male sex	0.97 (0.36–2.61)	0.953
Baseline NIHSS score	0.91 (0.82–1.02)	0.106
ASPECT value	0.78 (0.56–1.07)	0.120
Location of intracranial occlusion	0.89 (0.49–1.59)	0.690
Serum glucose	1.01 (1.01–1.02)	0.002
Neutrophil-to-lymphocyte ratio	1.11 (1.04–1.18)	0.001

ORs for every 1-point increase in age, NIHSS score, serum glucose, ASPECT value, neutrophil-to-lymphocyte ratio, and male sex are obtained with logistic regression analysis. * Adjustment for age, sex, baseline NIHSS score, ASPECT value, location of intracranial occlusion, serum glucose, and neutrophil-to-lymphocyte ratio. Abbreviations: ASPECT = Alberta Stroke Program Early CT, CI = confidence interval, NIHSS = National Institutes of Health Stroke Scale, OR = odds ratio.

## Data Availability

Anonymized data will be shared by request from any qualified investigator.
